# Identification of serum microRNAs as diagnostic biomarkers for schizophrenia

**DOI:** 10.1186/s41065-019-0099-3

**Published:** 2019-06-27

**Authors:** Kuanjun He, Chuang Guo, Meng Guo, Shuping Tong, Qiuli Zhang, Hongjun Sun, Lin He, Yongyong Shi

**Affiliations:** 1 College of Life Science, Inner Mongolia University for Nationalities, Tongliao, 028043 People’s Republic of China; 2 Network Center, Inner Mongolia University for Nationalities, Tongliao, 028043 People’s Republic of China; 3 Affiliated Hospital of Inner Mongolia University for Nationalities, Tongliao, 028043 People’s Republic of China; 4 Tongliao Institute of Mental Health, Tongliao, 028043 People’s Republic of China; 5 Bio-X Institutes, Key Laboratory for the Genetics of Developmental and Neuropsychiatric Disorders (Ministry of Education), Shanghai Jiao Tong University, Shanghai, 200030 People’s Republic of China; 6 Shanghai Key Laboratory of Psychotic Disorders, Shanghai Mental Health Center, Shanghai Jiao Tong University School of Medicine, Shanghai, 200030 People’s Republic of China; 7 Institute of Neuropsychiatric Science and Systems Biological Medicine, Shanghai Jiao Tong University, Shanghai, 200042 People’s Republic of China

**Keywords:** Schizophrenia, MiRNAs, Biomarkers, Identification, Serum

## Abstract

**Background:**

At present, the schizophrenia diagnoses are based on the clinical symptoms and behaviors neglecting the laboratory test indicators.

**Results:**

To better investigate the diagnostic potential of miRNAs for schizophrenia, we selected 14 candidate miRNAs and examined their expressions in the serums of 40 schizophrenia patients and 40 healthy controls by qRT-PCR. Ultimately three abnormally expressed microRNAs were identified, i.e., miR-34a-5p, miR-432-5p and miR-449a. Then, binary regression analysis was employed to combine 3 dysregulated miRNAs. ROC analysis revealed that the AUC of the combination of miR-432-5p + miR-449a in serums was 0.841 (95% CI: 0.791~0.887) with 90% sensitivity and 80% specificity. The AUC of the combination of miR-34a-5p + miR-432-5p + miR-449a in serums was 0.843 (95% CI: 0.791~0.887) with 90% sensitivity and 77.5% specificity. The results indicated that the combined model of miR-432-5p + miR-449a and miR-34a-5p + miR-432-5p + miR-449a have better prediction performances.

**Conclusions:**

The study concludes that the two miRNAs combinations have the potential to be used as biomarkers for schizophrenia diagnoses. The finding may be conducive to overcoming the dilemmas faced by current schizophrenia diagnosis.

## Background

Schizophrenia is one of the common kinds of mental diseases that has a lifetime risk of nearly 1% [1]_._ Currently, schizophrenia diagnoses are based on the clinical assessment of symptoms and behavior abnormalities of patients. The laboratory tests of pathophysiological and biochemical indicators are not available to assist the clinical diagnoses of schizophrenia. MiRNAs are a class of short endogenous non-coding RNA, which participate in the post-transcriptional regulation of protein-coding genes [2, 3]. It has been found that miRNAs regulate many essential biological functions and processes [4, 5] and are involved in almost all the life processes [6]. The changes of miRNA expressions reflect the alteration of neuropsychiatric disorders in the genetic and biological aspects [7, 8], including schizophrenia [9–11].

Although the causes of schizophrenia is still unclear, abnormally expressed miRNAs were detected in brain tissues [12–14], whole blood [15, 16], serum [17], plasma [18, 19] and peripheral blood mononuclear cells (PBMCs) [20, 21] of schizophrenia patients. These miRNAs may be used as the biomarkers for schizophrenia diagnoses. In fact, the biomarkers based on blood diagnostics and treatment has drawn increasingly attention [21–23]. Lai et al. (2011) discovered 7 abnormally expressed miRNAs, i.e., miR-34a, miR-564, miR-449a, miR-548d, miR-432, miR-652 and miR-572 in PBMCs of schizophrenia patients [21]. Shi et al. (2012) verified that 5 miRNAs, i.e., miR-181b, miR-1308, miR-219-2-3p, miRNA-195 and let-7 g expressed aberrantly in the schizophrenia patient serums [17]. Sun et al. (2015) analyzed 9 schizophrenia-related miRNAs in plasma and PBMCs of schizophrenia patients and miRNA30e of plasma was confirmed as a biomarker for the schizophrenia diagnosis [19]. Wei et al. (2015) verified that miR-193a-3p and miR-130b could be used as biomarkers for the schizophrenia diagnosis [24]. Sun et al. (2015) identified that miR-30e, miR-34a, miR-346, miR-181b, and miR-7 in the plasma could be non-invasive schizophrenia diagnostic markers [18]. With 7 miRNAs, i.e., miR-34a, miR-564, miR-449a, miR-548d, miR-432, miR-652 and miR-572 were confirmed as potential biomarkers for schizophrenia, Lai et al. (2016) verified again that the 7 miRNA expressions did not change in the peripheral blood of schizophrenia patients that hospitalized for more than two months and whose symptoms were alleviated, so their expressions were stable as diagnostic biomarkers of schizophrenia [16]. Liu et al. (2017) indicated that the axis of EGR1-miR-30a-5p-NEUROD1 could be used as diagnosis biomarker in an acute psychotic state of schizophrenia patients [25]. Ma et al. (2018) found miR-22-3p, miR-137, and miR-92a-3p expressed aberrantly in schizophrenia patient’s peripheral blood and the combination of miR-22-3p + miR-137 + miR-92a-3p could be used as a schizophrenia diagnosis biomarker [26].

Based on the above literature, we selected 14 candidate miRNAs that were reported as a possible biomarkers in the whole blood, plasma, PBMCs, or serums of schizophrenia patients. The 14 candidate miRNAs are miR-30e-5p, miR-130b-3p, miR-652-5p, miR-193a-3p, miR-181b-5p, miR-34a-5p, miR-346, miR-572, miR-7-5p, miR-449a, miR-564, miR-432-5p, miR-548d-3p, and miR-30a-5p. We used qRT-PCR to check the expressions of 14 miRNAs which expressed in the serums of 40 schizophrenia patients vs 40 healthy controls and further explored their diagnostic values and functions for schizophrenia.

## Materials and methods

### Participants

All the subjects of the study were diagnosed as schizophrenia patients by at least two experienced psychiatrists based on the criteria specified in the *Diagnosis and Statistical Manual of Mental Disorders* Fourth Edition (DSM-IV) and International Classification of Diseases 10 (ICD10). All the patients were treated with antipsychotic drugs including aripiprazole, risperidone, olanzapine, and clozapine. Control groups were recruited from volunteers. Blood donors and any individual who suffered or are suffering a mental illness and whose relatives suffered or are suffering with mental illness were excluded. Before conducting this study, we obtained the written consent from all the participants. The study was approved by the ethical committee of Tongliao Institute of Mental Health, Tongliao, China.

### Peripheral blood collection

Independent peripheral blood samples (5 ml per participant) was obtained from 80 subjects in the morning on an empty stomach and were collected in EDTA anticoagulant tubes. After standing for half an hour on ice, the supernatant was aspirated. 10,000 g Centrifuged for 5 min and discard the precipitate. The supernatant that left was stored at − 80 °C until using for extraction of total RNA.

### miRNA selection

We preliminarily selected 14 candidate miRNAs, i.e., miR-30e-5p, miR-130b-3p, miR-652-5p, miR-193a-3p, miR-181b-5p, miR-34a-5p, miR-346, miR-572, miR-7-5p, miR-449a, miR-564, miR-432-5p, miR-548d-3p, and miR-30a-5p in the whole blood, plasma, PBMCs or serums based on diagnostic potential confirmed by previous studies.

### RNA extraction and miRNA quantification by QRT-PCR

Total RNAs were harvested using miRcute Serum/Plasma miRNA Isolation Kit (TIANGEN BIOTECH, China) and quantified using a NanoDrop 2000 (Thermo, USA) according to the manufacturer’s instructions. Total RNA (1 μg) from each sample was reverse-transcribed using the miRcute Plus miRNA First-Strand cDNA Kit (TIANGEN BIOTECH, China). Quantitative real-time PCR was performed with a 2 × SYBR qPCR Mix (Beijing Zoman Biotechnology Co., Ltd., China) to quantify the miRNA expression. In order to normalize miRNA expression, the study selected U6 small nuclear RNA as internal reference. Each sample was detected three times, and the 2^-ΔΔCt^ method was used to analyze the expression data [27]. The primer information for the miRNAs and U6 is shown in Table 1.

**Table 1:** Forward primers of miRNAs and primers of U6

Name	Previous IDs	Accession number	Mature sequence	Sequence (from 5′to 3′)
hsa-miR-30e-5p	hsa-miR-30e	MIMAT0000692	UGUAAACAUCCUUGACUGGAAG	TGTAAACATCCTTGACTGGA
hsa-miR-130b-3p	hsa-miR-130b	MIMAT0000691	CAGUGCAAUGAUGAAAGGGCAU	CAGTGCAATGATGAAAGGGCAT
hsa-miR-193a-3p	hsa-miR-193a-3p	MIMAT0000459	AACUGGCCUACAAAGUCCCAGU	AACTGGCCTACAAAGTCCCAGT
hsa-miR-181b-5p	hsa-miR-181b	MIMAT0000257	AACAUUCAUUGCUGUCGGUGGGU	AACATTCATTGCTGTCGGTGGGTT
hsa-miR-34a-5p	hsa-miR-34a	MIMAT0000255	UGGCAGUGUCUUAGCUGGUUGU	TGGCAGTGTCTTAGCTGGTTGTT
hsa-miR-346	hsa-miR-346	MIMAT0000773	UGUCUGCCCGCAUGCCUGCCUCU	TGCCCGCATGCCTGCCTCT
hsa-miR-7-5p	hsa-miR-7	MIMAT0000252	UGGAAGACUAGUGAUUUUGUUGUU	TGGAAGACTAGTGATTTTGTT
hsa-miR-449a	hsa-miR-449	MIMAT0001541	UGGCAGUGUAUUGUUAGCUGGU	TGGCAGTGTATTGTTAGCTGGT
hsa-miR-564	hsa-miR-564	MIMAT0003228	AGGCACGGUGUCAGCAGGC	AGGCACGGTGTCAGCAGGC
hsa-miR-432-5p	hsa-miR-432	MIMAT0002814	UCUUGGAGUAGGUCAUUGGGUGG	TCTTGGAGTAGGTCATTGGGTGG
hsa-miR-548d-3p	hsa-miR-548d	MIMAT0003323	CAAAAACCACAGUUUCUUUUGC	GCCAAAAACCACAGTTTCTTTTGC
hsa-miR-572	hsa-miR-572	MIMAT0003237	GUCCGCUCGGCGGUGGCCCA	CGCTCGGCGGTGGCCCA
hsa-miR-652-5p	hsa-miR-652	MIMAT0022709	CAACCCUAGGAGAGGGUGCCAUUCA	CAACCCTAGGAGAGGGTGCCATTCA
hsa-miR-30a-5p	hsa-miR-30a-5p	MIMAT0000087	UGUAAACAUCCUCGACUGGAAG	CACTCTCATGTAAACATCCTCGAC
U6 snRNA (Forward)		NR_004394		CTCGCTTCGGCAGCACA
U6 snRNA (Reverse)				AACGCTTCACGAATTTGCGT

### Statistical analyses

Data were analyzed using SPSS software version 20.0 (New York, USA), GraphPad Prism version 7.0 (California, USA) and MedCalc Statistical Software version 18.2.1 (MedCalc Software bvba, Ostend, Belgium; http://www.medcalc.org; 2018). Data (presented as mean ± SEM) were analyzed using the Mann-Whitney U test. Statistically significant level was set at *P*-values < 0.05. Using binary logistic regression to combine of 3 dysregulated miRNAs in the regression equation. The diagnostic performances of three miRNAs were evaluated by ROC curves.

## Results

**Fig. 1:**
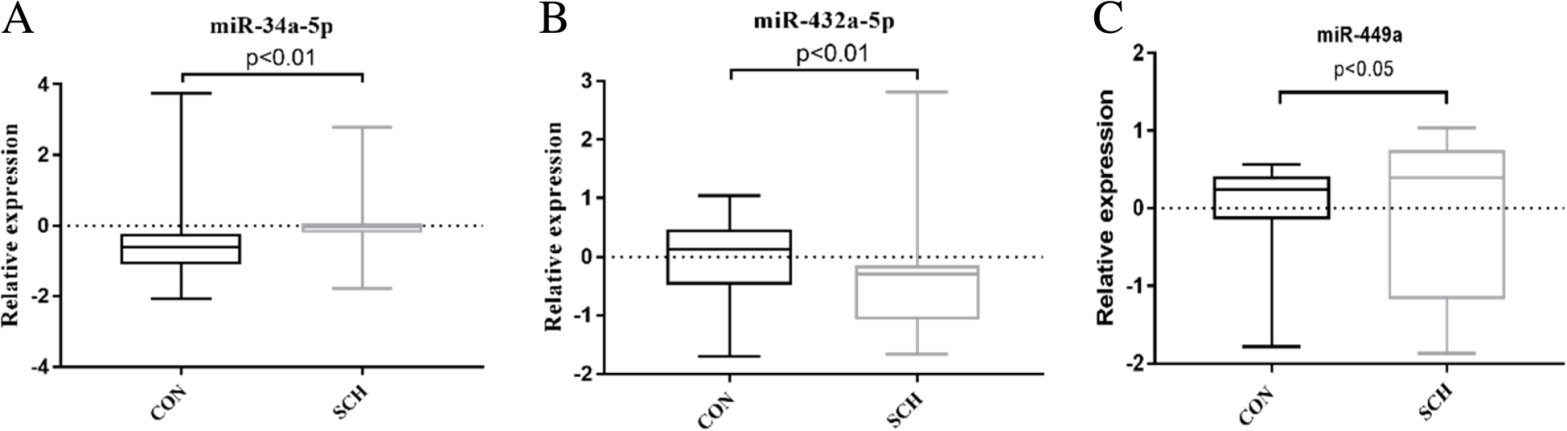
Discrimination of 3 miRNAs levels in serums of SCH and CON. The y axis indicates log_10_2^-ΔΔCt^ relative expression of aberrant miRNAs of quantitative real-time PCR. The expression of 3 miRNAs of 14 miRNAs was significantly different in 40 SCH than in 40 CON. (**a**) miR-34a, (**b**) miR- 432-5p, (**c**) miR-449a. The plots were constructed using GraphPad Prism 7 software and statistical difference was analyzed by Mann-Whitney U test. CON, healthy controls; SCH, schizophrenia patients

Baseline demographic characteristics of the patient and control groups are shown in Table 2. No significant difference is found in age and gender between the two groups (all *p* > 0.05). QRT-PCR was performed to compare the expression differences of the 14 candidate miRNAs between patients and controls. The expressions of miR-34a-5p, miR-432-5p and miR-449a in schizophrenia patients were significantly different from those of the healthy control. Relative levels of the 3 significantly altered miRNAs can be seen in Fig. 1.

**Table 2:** The demographics of all subjects

	Patients	Healthy controls	F/χ2	p
Age (years)	30.35 ± 6.367	27.9 ± 7.873	2.342	0.232
Gender (M/F)	22/18	19/21	0.45	0.502

**Fig. 2:**
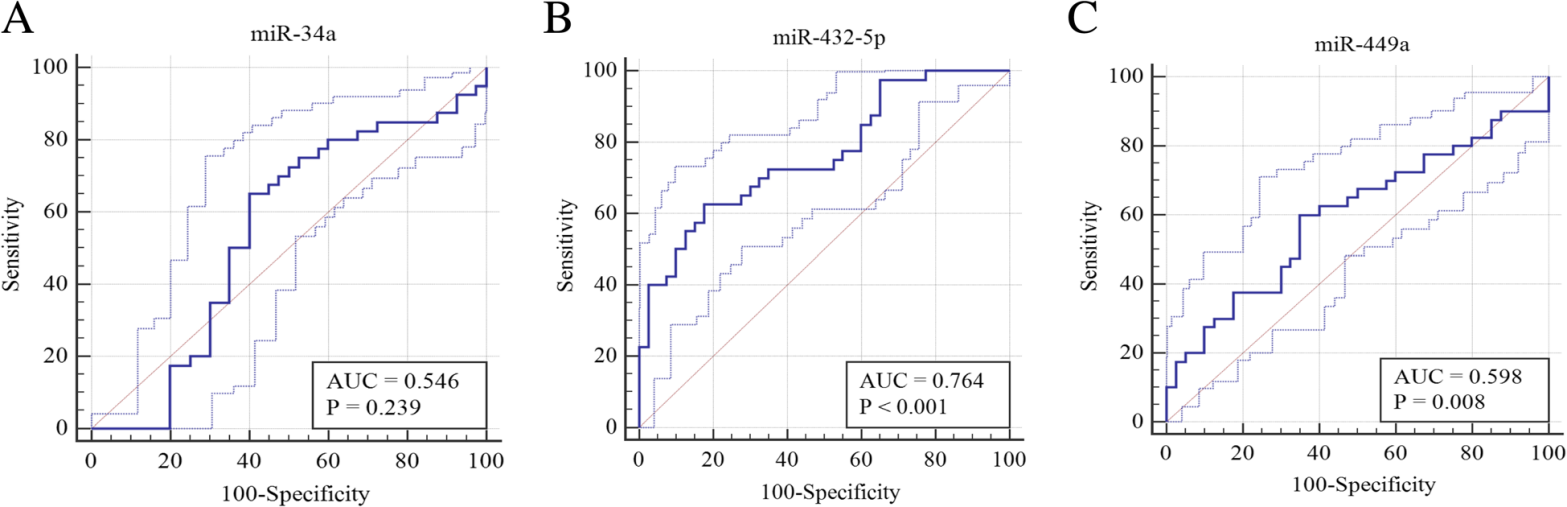
ROC analysis for the miR-34a, miR-432-5p, and miR-449a (**a**) miR-34a (**b**) miR-432-5p (**c**) miR-449a.

**Fig. 3:**
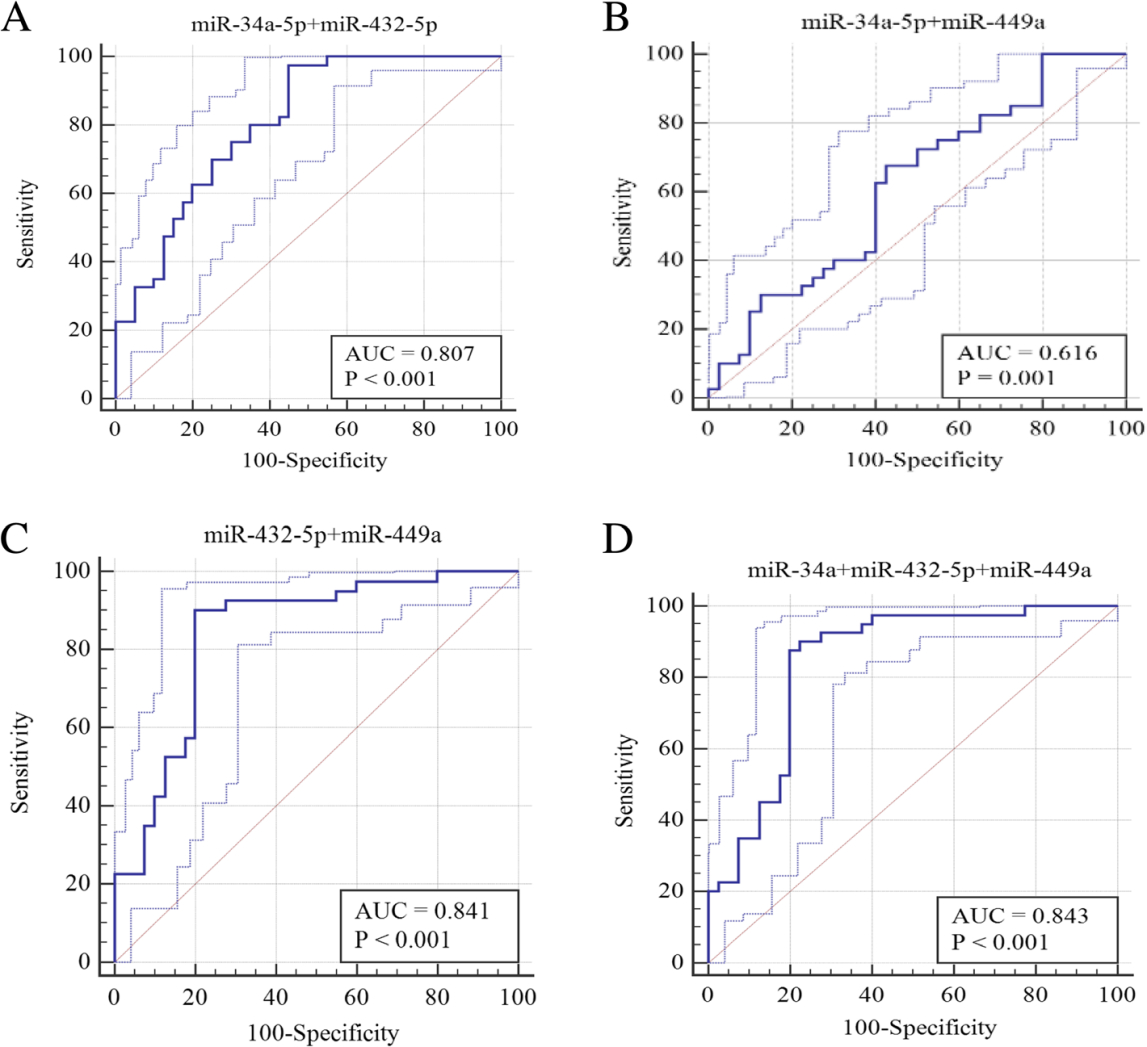
ROC curves for diagnostic models of the combination of miR-34a-5p + miR-432, miR-34a-5p + miR-449a, miR-432-5p + miR-449a, miR-34a-5p + miR-432-5p + miR-449a (**a**) The combination of miR-34a-5p + miR-432 (**b**) The combination of miR-34a-5p + miR-449a (**c**) The combination of miR-432-5p + miR-449a. (**d**) The combination of miR-34a-5p + miR-432-5p + miR-449a.

ROC curves of the 3 miRNAs were constructed to further evaluate their diagnostic values. As shown in Fig. 2 and Table 3, miR-432a-5p has the best discriminatory performance with an AUC of 0.764 (95% CI: 0.706~0.817), 62.5% sensitivity, 82.5% specificity and 0.45 Youden index. The ROC curves demonstrated that the combination of miR-34a-5p + miR-432-5p, miR-432-5p + miR-449a, miR-34a-5p + miR-432-5p + miR-449a had a better discriminatory performance with a respective AUC of 0.807 (95%CI: 0.752~0.855), 0.841 (95%CI: 0.789~0.885) and 0.843 (95%CI: 0.791~0.887) (See Fig. 3 and Table 3). The combination of miR-34a-5p + miR-432-5p has 97.5% sensitivity, 55% specificity and 0.525 Youden index J; the combination of miR-432-5p + miR-449a has 90% sensitivity, 80% specificity and 0.7 Youden index J; the combination of miR-34a-5p + miR-432-5p + miR-449a has 90% sensitivity, 77.5% specificity and 0.675 Youden index J (See Table 3). In terms of the indicators such as sensitivity, specificity and Yoden index, the combination of miR-432-5p + miR-449a and the combination of miR-34a-5p + miR-432-5p + miR-449a display a better discriminatory performance.

**Table 3:** Diagnostic accuracy based on serum miRNA levels

miRNAs	AUC	95%CI	P-value	P-Bonferroni	Sensitivity	Specificity	Youden index J
miR-34a-5p	0.546	0.480~0.610	0.2392	3.3488	65%	60%	0.25
miR-432-5p	0.764	0.706~0.817	< 0.0001	< 0.01	62.50%	82.50%	0.45
miR-449a	0.598	0.533~0.661	0.008	0.112	60.00%	65.00%	0.25
miR-34a + miR-432-5p	0.807	0.752~0.855	< 0.0001	< 0.01	97.50%	55.00%	0.525
miR-34a + miR-449a	0.616	0.552~0.678	0.0014	0.0196	67.50%	57.50%	0.25
miR-432-5p + miR-449a	0.841	0.789~0.885	< 0.0001	< 0.01	90.00%	80.00%	0.7
miR-34a + miR-432-5p + miR-449a	0.843	0.791~0.887	< 0.0001	< 0.01	90.00%	77.50%	0.675

## Discussion

The current study selected 14 candidate miRNAs and examined their expressions in the serums to explore their diagnostic value for schizophrenia. The qRT-PCR result showed that the expressions of miR-34a-5p, miR-432-5p, and miR-449a have difference in schizophrenia patients. The AUC of the combinations of miR-432-5p + miR-449a and miR-34a-5p + miR432-5p + miR-449a were greater compared with any single miRNA, indicating that the two combination of miRNAs can be used as biomarkers for schizophrenia.

The study confirmed that miR-34a, miR-432, and miR-449a expressed significant changes in serums of schizophrenia patients, suggesting that they may play an important role in the development of schizophrenia. MiR-34a has some well-documented involvements in neurogenesis and neural differentiation [28, 29]. The change of the expression of miR-34a has been found in brain tissues of patients with psychiatric disorder [30, 31]. Schipper et al. (2007) found that the expression of miR-34a significantly up-regulated in the PBMCs of patients with Alzheimer’s disease [32]. The expression level of miR-432 was observed to alter in postmortem cerebellar cortex from autism patients [33]. The miR-449 expression level was found deregulated in cerebrospinal fluid of AD patient brains [34]. Wu et al. (2014) verified that miR-449 and miR-34b/c are very important in normal brain development [35]. Using the results of genome-wide association analysis (GWAS) of schizophrenia, Hauberg et al. predict miRNAs target genes and performed regression and enrichment analysis to explore the regulation of miRNAs on schizophrenia risk genes, they listed 10 conserved miRNAs in the enrichment analysis, including miR-34 ac-5p and miR-449a, and indicated these miRNAs play an important role in schizophrenia [36]. In addition, several target genes of hsa-miR-34a were found using the predictions of TargetScan, such as GREM2, TANC2, CAMSAP1, RGMB, CALN1, RTN4RL1 and FKBP1B are related to the development and function of neuron [16]. The study showed that miR-34a-5p, miR-432-5p, and miR-449a expressed aberrantly in the serums of schizophrenia patients. Before then, Sun et al. (2015) verified the abnormal expression of miR-34a existed in PBMCs of schizophrenia patients [19]. Sun et al. (2015) found again the expressions of miR-34a and miR-432 exhibited the difference in plasma [18]. Yu et al. (2014) verified that the expression level of miR-432 were significantly down-regulated in PBMCs of schizophrenia patients before treatment compared with healthy controls [23]. Lai et al. (2016) verified that the miR-34a, miR-432 and miR-449a did not change in the PBMCs of patients that hospitalized for more than two months and whose symptoms were alleviated [16]. They believe the hospitalization and symptom alleviation could not affected the expressions of circulating miRNAs, so they were sufficiently stable and detectable biomarkers in peripheral blood [16]. In addition, Lai et al. (2014) investigated the expression changes of blood-based miRNA from preterm infants to adulthood, and found miR-34a, miR-432, and miR-449a were expressed consistently from infancy to adulthood [37].

The limitations of the study includes the lack of distinction among psychotic symptoms and the limited number of subjects. All the selected patients in the current study were treated with antipsychotic drugs. We can not exclude the possibility of the expressions of change in these miRNAs before and after the treatment.

In conclusion, we check the expressions of 14 candidate miRNAs in the serums of 80 subjects and found that miR-34a-5p, miR432-5p, and miR-449a expressed aberrantly in schizophrenia patients. The AUC value indicated that the combination of miR-432-5p + miR-449a and all three miRNAs could be used as biomarkers for schizophrenia. We propose that two novel schizophrenia diagnostic model presented here can be treated as biomarkers for schizophrenia and they are useful for overcoming the limitations faced by current schizophrenia diagnoses.

## Data Availability

The original datasets are available from the corresponding author on reasonable request.

## Abbreviations

AUC: Area under the curve
DSM-IV: Diagnosis and Statistical Manual of Mental Disorders Fourth Edition
ICD10: International Classification of Diseases 10
MiRNAs: MicroRNAs
QRT-PCR: Quantitative real-time polymerase chain reaction
ROC: Receiver operating characteristics
